# Combination Therapies for the Treatment of Advanced Melanoma: A Review of Current Evidence

**DOI:** 10.1155/2014/307059

**Published:** 2014-02-12

**Authors:** Mark Voskoboynik, Hendrik-Tobias Arkenau

**Affiliations:** Sarah Cannon Research Institute UK, London W1G 6AD, UK

## Abstract

The treatment of advanced melanoma has been revolutionised in recent years with the advent of a range of new therapies. BRAF inhibitors, such as vemurafenib, have demonstrated improvements in the overall survival of patients with advanced melanoma that harbour a BRAF V600 mutation. Alongside these targeted therapies, novel immune-checkpoint inhibitors, such as ipilimumab, have also been developed and have produced similarly improved outcomes for patients. For the first time in the history of melanoma, monotherapy with each of these drugs has produced improvements in the overall survival of patients with advanced disease. Building on this initial success, there has been intense interest in developing combination therapies predominantly with either dual blockade of the MAPK oncogenic pathway or dual immune-checkpoint blockade. The current evidence for the use of these combination therapies will be presented here.

## 1. Introduction

Melanoma is the fifth most common malignancy in males and the sixth in women [1]. Enormous advances in the treatment of melanoma have occurred in recent years with an improved understanding of the molecular pathways driving this malignancy as well as the critical importance of the immune system in this process. These therapeutic advances have provided the foundations for further improvements in patient outcomes. The initial studies have demonstrated improvements in survival in a cancer that has previously been shown to be chemotherapy resistant but they have also revealed some limitations. The shortfalls are either due to a short duration of response because of resistance or due to significant treatment related toxicity. There are currently significant efforts being made not only to further understand resistance but also to improve treatments with newer drugs and more importantly, which is the focus of our review, rational use of combination therapy. In the footsteps of Professor Frei III and colleagues who introduced the concept of combination chemotherapy to improve patient outcomes, modern oncologists and researchers are developing rational combinations of novel targeted therapies and immunotherapies to both improve patient outcomes and reduce toxicity [2]. Our review will update current evidence for combination targeted therapies and immunotherapies for the treatment of advanced melanoma.

## 2. MAPK Pathway Inhibition

**Figure 1:**
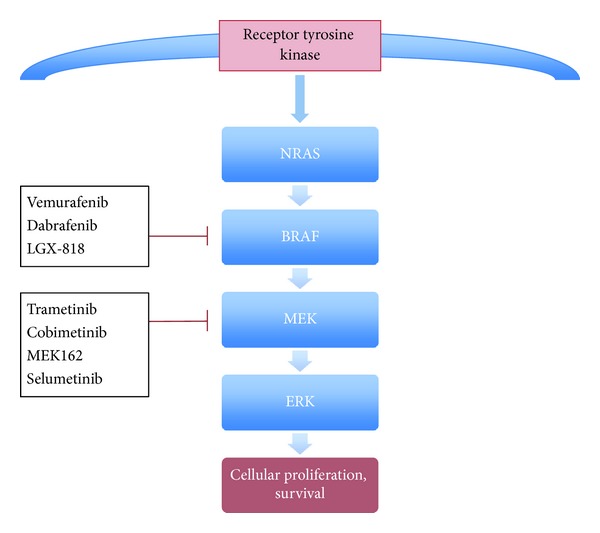
The MAPK signalling pathway and drugs currently in development.

The discovery that more than 65% of melanomas contain activating mutations of the RAS/RAF/MEK/ERK pathway made this pathway a key focus of drug development in melanoma (see Figure 1) [3]. Mutations in the BRAF kinase are the most commonly identified, seen in between 40 and 50% of cutaneous melanomas, in particular at the V600 position [4]. A further 10 to 15% of melanomas have the mutually exclusive NRAS mutation, another important driver mutation in melanoma [5, 6].

The efficacy and survival advantage of BRAF inhibitor monotherapy has been demonstrated in several clinical studies [10, 11]. In a landmark phase 3 study by Chapman and colleagues, vemurafenib monotherapy showed an overall survival advantage when compared to dacarbazine in treatment-naïve patients with advanced BRAF V600E mutated melanoma [10]. At 6 months, overall survival was 84% (95% confidence interval [CI], 78 to 89) in the vemurafenib group and 64% (95% CI, 56 to 73) in the dacarbazine group. Subsequent analyses by Chapman et al. with longer followup showed that the median survival with vemurafenib and dacarbazine was 13.2 months (95% CI 12.0–15.0) and 9.6 months (95% CI 7.9–11.8), respectively [16]. Despite demonstrating a clear improvement, the PFS was still only 5.3 months (vemurafenib) compared to 1.6 months (dacarbazine). Median survival of previously treated patients that received vemurafenib as part of a phase 2 trial was approximately 16 months [17]. 

Another BRAF inhibitor, dabrafenib has also been compared with dacarbazine in a phase 3 study of previously untreated advanced melanomas and this positive study showed an improvement in PFS, the study's primary endpoint. The median PFS was 5.1 months for dabrafenib and 2.7 months for dacarbazine, with a hazard ratio (HR) of 0.30 (95% CI 0.18–0.51; *P* < 0.0001) [11]. An updated analysis was presented in abstract form by Hauschild and colleagues [18]. With longer followup, the median PFS was 6.9 months (dabrafenib) and 2.7 months (dacarbazine). The OS results, although in favour of dabrafenib (18.2 months versus 15.6 months), were not statistically significant probably because more than half of the patients on dacarbazine crossed over to receive dabrafenib at progression.

The most common toxicity seen with both of these BRAF inhibitors was their cutaneous effects. Photosensitivity and various hyperproliferative skin disorders including keratoacanthomas and cutaneous squamous cell carcinomas were the most commonly seen, especially in older patients with more chronically sun-damaged skin. The most likely explanation for this is that the BRAF inhibitor causes paradoxical activation of MEK in normal cells [19].

Overall, we can see that a consistent median PFS of approximately 6 months has been demonstrated in these BRAF inhibitor monotherapy studies. So, despite large advances over the previous standard of care, there is still clearly significant room for improvement to overcome fairly early resistance to BRAF inhibitor monotherapy. A number of potential mechanisms for resistance have been identified. The key rationale for combining BRAF and MEK inhibitors is to overcome downstream reactivation of MEK signaling [20]. Other potential acquired mechanisms of resistance are activation of other oncogenic pathways such as the PI3 K/AKT/mTOR pathway [21–23]. This review will focus on the combination that is most advanced in clinical studies, the combination of BRAF and MEK inhibitors.

### 2.1. Combination Blockade of the MAPK Pathway

Flaherty et al. published the results of a phase 1/2 study that investigated the combination of dabrafenib and trametinib, a MEK1 inhibitor [7]. Between 2010 and 2011 they enrolled 247 patients with advanced melanoma harbouring either V600E or V600K mutations. Data from 162 patients in the phase 2 component of the study was presented. Patients were randomised to one of three arms: either a combination of dabrafenib 150 twice daily (BD) and trametinib 1 mg daily or dabrafenib 150 mg BD and trametinib 2 mg daily or dabrafenib 150 mg as monotherapy. Patients who progressed on the dabrafenib monotherapy arm were allowed to crossover to a combination treatment. After a median followup of 14 months, patients on the combination arm with 2 mg trametinib had a median progression free survival (PFS) of 9.4 months compared to 5.8 months in the dabrafenib monotherapy arm (HR 0.39, *P* < 0.001). In fact, at 12 months after therapy was commenced, 41% of the patients on the combination (trametinib 2 mg) arm were alive and progression free compared with only 9% of the monotherapy arm (*P* < 0.001). The improvements in PFS were seen in both V600E and V600K patients. Combination therapy patients (trametinib 2 mg) also had an increased duration of response compared to monotherapy patients (10.5 months versus 5.6 months). Importantly, the combination therapy caused less toxicity than the monotherapy, in particular cutaneous toxicity. Cutaneous squamous cell carcinomas (SCC) and keratoacanthomas were identified in 19% of monotherapy patients compared with between 2% and 7% of combination therapy patients. Specific MEK-inhibitor related side effects, including peripheral oedema, hypertension, and ocular events, were more common in the combination therapy arms. This study therefore demonstrated that the dabrafenib and trametinib combination improves clinical outcomes and reduces toxicity compared to dabrafenib monotherapy. It confirms that dual blockade delays development of acquired BRAF inhibitor resistance.

Sosman et al. presented, in abstract form, data from the same study as above [24]. This study compared the efficacy of the dabrafenib and trametinib combination between BRAF inhibitor resistant and BRAF inhibitor naïve patients. The 69 patients in the BRAF inhibitor resistant group had an overall response rate (ORR) of 9–15% compared to an ORR of 63–76% in the 78 BRAF inhibitor naïve patients. This study shows that it is far more effective to start with dual blockade therapy upfront rather than delaying it until after BRAF resistance has occurred.

Preliminary results from a phase 1b/2 study of the combination of LGX818, a potent BRAF inhibitor, and MEK162, a selective MEK 1/2 inhibitor, have been presented by Kefford et al. [8]. At the time of the interim results, 20 patients with BRAF V600-dependent advanced solid tumours had been treated. Both BRAFi naïve and pretreated patients were included. No photosensitivity, SCC, hyperkeratosis, or hand-foot syndrome was seen suggesting good treatment tolerability. Thus far, 1 of 7 patients (14%) with at least 1 postbaseline scan in the BRAFi naïve group has had a complete response. Five of these 7 patients had a partial response. Conversely, only 2 of 9 (22%) BRAFi pretreated patients had a partial response. These results seem to be consistent with the studies by Sosman et al. and Flaherty et al. in that combination therapy, for BRAFi naïve patients, appears more effective and better tolerated.

Vemurafenib, a BRAFi with proven efficacy as a single agent, is being studied in combination with GDC-0973, a MEK inhibitor. Preliminary data from this phase 1b study (BRIM 7) was presented by Gonzalez et al. [9]. Analyses of 44 patients, treated in this study, with advanced BRAF V600 mutated melanoma that are either vemurafenib naïve or previously progressed on vemurafenib were presented. All eight of the vemurafenib naïve patients that have received treatment have had tumour reduction thus far.

### 2.2. Ongoing Studies

There are at least 2 other ongoing trials that are comparing the combination of BRAF and MEK inhibitor therapy to BRAF inhibitor monotherapy. The first is an open label phase 3 looking at dabrafenib plus trametinib versus vemurafenib alone in unresectable or metastatic BRAF V600E/K cutaneous melanoma (ClinicalTrials.gov, NCT01597908). The second is a double blinded randomized phase 3 study comparing trametinib and dabrafenib combination therapy to dabrafenib monotherapy in subjects with BRAF-mutant melanoma (ClinicalTrials.gov, NCT01584648). 

Overall, the BRAF/MEK inhibitor combination is proving to be not only more effective but also better tolerated than each agent delivered alone (see Table 1). This combination therapy therefore appears a very promising treatment option for BRAF mutant melanomas and is likely to play a pivotal role in the treatment of this group of patients.

## 3. Immune-Checkpoint Blockade

**Figure 2:**
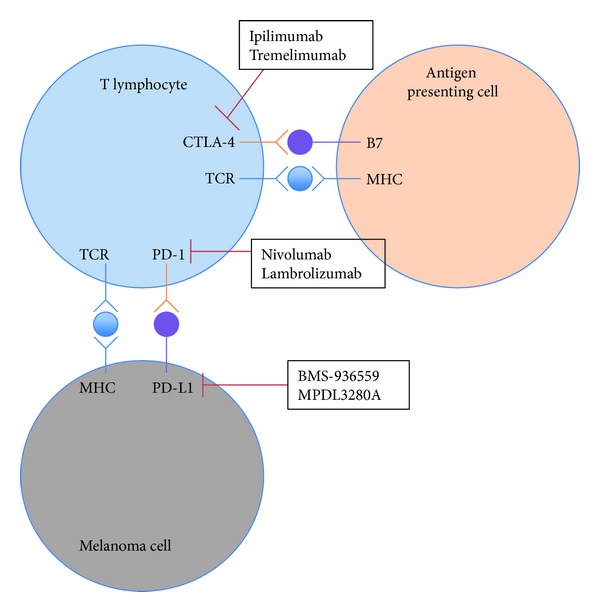
Important immune-checkpoint interactions and relevant inhibitory antibodies in current development.

Immunotherapy has long been investigated as a therapy for advanced melanoma from early attempts to induce an immune response by intratumoral injection of BCG to injections of viruses and many vaccines. High dose IL-2 therapy has been studied for more than 20 years and, although it has never been demonstrated to improve overall survival, a distinct minority of patients may achieve a durable response [25, 26]. It was approved by the FDA in 1998 for use in metastatic melanoma and has been included in the recently published Society for Immunotherapy of Cancer consensus statement on tumour immunotherapy for the treatment of cutaneous melanoma [27]. Immune-checkpoint blockade, predominantly targeting cytotoxic T-lymphocyte-associated antigen 4 (CTLA-4) and PD-1/PD-L1-is currently the main focus of immunotherapies in metastatic melanoma (see Figure 2).

CTLA-4 is a molecule that is important for downregulating pathways of T cell activation. Blockade of this immune-checkpoint molecule with monoclonal antibodies such as ipilimumab and tremelimumab has been examined.

Two landmark phase 3 trials have demonstrated an improvement in overall survival with ipilimumab in patients (previously treated and untreated) with advanced melanoma [12, 13]. In 676 patients that had previously been treated with either IL-2 or cytotoxic chemotherapy, ipilimumab alone improved the median overall survival compared to gp100 (10.1 months versus 6.4 months, HR 0.66; *P* = 0.003) [12]. In a second trial, 502 previously untreated patients received ipilimumab plus dacarbazine or placebo plus dacarbazine [13]. The group receiving ipilimumab and dacarbazine had an improved median overall survival compared with the placebo/dacarbazine group (11.2 versus 9.1 months).

In these two studies objective response rates to ipilimumab were consistently low (10.9 percent to 15 percent) but seemed durable, particularly in the minority of patients that achieved a complete response to therapy. This has also been demonstrated by Prieto and colleagues when examining the long-term followup of 177 patients with metastatic melanoma with 14 of the identified 15 complete responders continuing to respond at more than 54 months [28].

The improvements in overall survival seen with ipilimumab came at a cost of significant rates of immune related toxicity. Between 15 and 20% of patients receiving ipilimumab experienced clinically significant autoimmune adverse effects, most commonly dermatological, gastrointestinal, and endocrine (thyroid, pituitary, and adrenal). When ipilimumab was given in combination with dacarbazine even higher rates of immune related adverse events were seen, largely due to the increased rates of hepatic toxicity.

A phase 3 randomized clinical trial that compared tremelimumab with standard-of-care chemotherapy in chemotherapy naïve patients with advanced melanoma failed to show a statistically significant survival advantage [29]. 655 patients participated in the study and received either tremelimumab or chemotherapy (dacarbazine or temozolomide). The median overall survival was 12.6 months for tremelimumab and 10.7 months for chemotherapy but this was not statistically significant (hazard ration, 0.88; *P* = 0.127). Although response rates between the two arms were similar (10.7% in the tremelimumab arm and 9.8% in the chemotherapy arm) the response duration was significantly longer after tremelimumab (35.8 versus 13.7 months; *P* = 0.0011).

Inhibition of programmed cell death-1 (PD-1) and its primary ligand, PD-L1, has recently been shown to have efficacy in a number of cancers, including melanoma. PD-1 is a receptor expressed by T cells and PD-L1 is its ligand that is expressed on tumour cells. PD-1/PD-L1 antibodies are different in their immune activation to CTLA-4 inhibitors as they attempt to improve the antitumour T cell response in a more specific, tumour-directed manner.

A number of early studies of PD-1 inhibitors (nivolumab and lambrolizumab) and PD-L1 inhibitors (BMS-936559, MPDL3280A) have been reported or are ongoing [14, 30–32]. Early experience with nivolumab, a fully human IgG4 antibody blocking PD-1 showed that it results in objective response rates of about 30% and these responses were often durable, over a year in duration. Immune related toxicities, similar to that experienced with CTLA-4 inhibitors, were seen with nivolumab but were less clinically significant. Pneumonitis was the most significant toxicity seen and had been the cause of treatment related deaths.

### 3.1. Dual Immune-Checkpoint Blockade

Combination therapy with ipilimumab and nivolumab has been assessed in a phase 1 trial by Wolchock and colleagues [15]. The rationale for their study was that each drug had a distinct immunological mechanism of action and a potential for improved clinical activity using the combination. This was a dose escalation study and 53 patients with advanced melanoma were treated in the concurrent therapy cohort. Both drugs were administered intravenously once every three weeks for four doses, followed by nivolumab alone every 3 weeks for 4 doses. Following this, the combined treatment was administered every 12 weeks up to 8 doses. In a separate, sequential cohort, 33 patients that had previously been treated with ipilimumab received nivolumab every 2 weeks for up to 48 doses.

In the concurrent cohort, 40 percent of evaluable patients had an objective response with 31 percent achieving at least an 80 percent reduction in tumour burden. In preliminary data, these responses appeared durable with 65 percent of patients demonstrating stable disease or greater for at least 24 weeks. By comparison, in the sequenced therapy cohort, 20 percent had an objective response.

93 percent of patients in the concurrent cohort experienced treatment related adverse events with the most commonly seen including rash, pruritus, fatigue, and diarrhoea. Grade 3 or 4 therapy related toxicity was seen in 53 percent of cases including hepatic (15%), gastrointestinal (9%), and renal (6%). Most of this toxicity was reversible.

This study further emphasises the improvements that can be made with rational combination therapies when measured in terms of response and survival and all with tolerable toxicity (see Table 2).

### 3.2. Ongoing Studies

There are a number of ongoing clinical trials that seek to further clarify the efficacy of combination immune-checkpoint blockade in patients with advanced melanoma. A phase 3 trial of nivolumab or nivolumab plus ipilimumab versus ipilimumab alone in previously untreated advanced melanoma has recently opened and is recruiting (ClinicalTrials.gov Identifier: NCT01844505). Nivolumab in combination with ipilimumab versus ipilimumab alone in treatment naïve advanced melanoma patients is also the subject of a randomized phase 2 trial (ClinicalTrials.gov Identifier: NCT01927419).

## 4. The Future

Combining active therapies to overcome resistances is key to making further advances in the treatment of metastatic melanoma. As always, with each key development or discovery, further questions arise. Sequencing of MAPK pathway inhibition and checkpoint blockade is clearly important although the best approach has not yet been studied but will no doubt be the subject of future studies. The possibility of combining the infrequent durable responses seen with immune-checkpoint blockade and the rapid, frequent but short-lived responses seen with MAPK inhibition is an exciting one. This is already the subject of a number of ongoing studies, a sample of which includes a phase 1/2 study examining the combination of vemurafenib and ipilimumab (ClinicalTrials.gov, Identifier: NCT01400451), a phase 1 study of dabrafenib +/− trametinib in combination with ipilimumab (ClinicalTrials.gov, Identifier: NCT01767454), and a four-armed phase 1 study of ipilimumab +/− dabrafenib and/or trametinib (ClinicalTrials.gov, Identifier: NCT01940809).

Other combinations between MAPK pathway inhibitors and other important oncogenic pathways (such as PI3K/AKT/mTOR) are also being investigated with at least 3 such trials already underway combining vemurafenib with PI3K inhibitors (BKM120, PX-866, and SAR260301) in phase 1 and 2 studies (ClinicalTrials.gov, Identifiers: NCT01512251, NCT01616199, and NCT01673737). A novel AKT inhibitor (MK2206) is being combined with the MEK inhibitor selumetinib in a National Cancer Institute sponsored phase 2 study (ClinicalTrials.gov, Identifier: NCT01519427).

A significant potential issue when developing novel drug combinations is that of unexpected increases in adverse events because of either overlapping toxicities or unpredictable drug-drug interactions. In their brief correspondence, Ribas and colleagues emphasise this same point, citing their phase 1 study of the concurrent administration of vemurafenib and ipilimumab [33]. A majority of the patients that they treated, in two dose levels, experienced grade 2 or 3 liver toxicity. The authors appropriately reinforce the need for carefully conducting trials of new combinations because of the unpredictable toxicities that might be identified. With newer drugs and even newer combinations, particular care must be taken in the early identification of toxicities, especially immune-related toxicities that can have disastrous consequences when not identified in a timely and appropriate way by experienced clinicians.

With this intense and promising drug development in metastatic melanoma, patient selection and individualizing treatments will become increasingly important as the therapeutic options continue to increase. Predictive biomarkers will be required to better target these drugs and to partly mitigate the escalating costs of these newer therapies. The challenges associated with an increase in treatment options is certainly a welcome one for oncologists accustomed to treating patients with advanced melanoma and this is undoubtedly only the start of a new era in the treatment of melanoma.

## Figures and Tables

**Table 1:** Comparison between the major trials of BRAF inhibitor monotherapy and BRAF/MEK inhibitor combination trials.

Drug + target	Study (author, date)	Patient number	Phase	Regimen + drugs (doses)	Response rate (%) (CR or PR)	Response duration	Drug related toxicity G3/4 AEs	Treatment related mortality	Median PFS (months)	OS (months)
Dabrafenib (D) + trametinib (T) BRAFi + MEKi	Flaherty et al., 2012 [7]	247 (162 in part C: combination (2 doses) versus monotherapy)	1/2	Part C groups: D (150 mg bd) + T (2 mg od) versus D (150 mg bd) + T (1 mg od) versus D (150 mg bd)	41 (*P* = 0.03) 27 (*P* = 0.77) 29	10.5 mo 9.5 mo 5.6 mo	58% 48% 43%	Nil Nil Nil	150/2: 9.4 (*P* < 0.001) 150/1: 9.2 (*P* = 0.006) 150: 5.8	NR

LGX818 + MEK162 BRAFi + MEKi	Kefford et al., 2013 [8]	20 (only 7 with at least one postbaseline scan in BRAF naïve group)	1b/2	LGX818 + MEK162 BRAFi naïve and BRAFi pretreated cohorts	Rx naïve: 86% Pretreated: 22%	NR	NR	NR	NR	NR

Vemurafenib + GDC-0973 BRAFi + MEKi	Gonzalez et al., 2012 [9]	44	1	Vemurafenib + GDC-0973 Vem. naïve and vem. pretreated groups	Vem. naïve: 100% Pretreated: NR	NR	NR	NR	NR	NR

Vemurafenib versus dacarbazine	Chapman et al., 2011 [10]	675	3	Vemurafenib (960 mg bd) versus dacarbazine (1000 mg/m^2^ q3w) Previously untreated	48 5	NR	NR	NR	5.3 1.6	NR (13.2)^a ^ NR (9.6)^a^

Dabrafenib versus dacarbazine	Hauschild et al., 2012 [11]	250	3	Dabrafenib (150 mg bd) versus dacarbazine (1000 mg/m^2^ q3w) Previously untreated		NR	53%^b^ 44%^b^	NR	5.1 (*P* < 0.0001) 2.7	NR (18.2)^c^ NR (15.6)^c^

^a^Updated median overall survival presented at a later updated analysis. ^b^Grade 2 toxicity or higher. ^c^Presented later at an updated analysis. Difference in median OS was not statistically significant.

**Table 2:** A comparison between CTLA-4 inhibitor monotherapy studies and the combination study of ipilimumab and nivolumab.

Drug + target	Study (author, date)	Patient number	Phase	Regimen + drugs (doses)	Response rate (%) (CR or PR)	Response duration	Drug related toxicity G3/4 AEs	Treatment related mortality	PFS (months)	OS (months)
Ipilimumab CTLA-4	Hodi et al., 2010 [12] Previously treated	676	3	Ipilimumab (3 mg/kg) versus ipilimumab + gp100 versus gp100	10.9 5.7 1.5	60% >26.5 months 17% >27.9 months 0% >2 years	22.9% 17.4% 11.4%	3.1% 2.1% 1.5%	2.86 2.76 2.76	10.1 10.0 6.4

	Robert et al., 2011 [13] Previously untreated	502	3	Ipilimumab (10 mg/kg) + dacarbazine versus dacarbazine	15.2 10.3	19.3 months (median) 8.1 months (median)	56.3% 27.5%	NR	<3 <3	11.2 9.1

Lambrolizumab CTLA-4	Hamid et al., 2013 [14]	655	1	Lambrolizumab	38	81% still responding at 11-month follow-up	13%	NR	>7	Not reached

Ipilimumab + nivolumab CTLA-4 + PD-1	Wolchok et al., 2013 [15]	86	1	Ipilimumab + nivolumab (concurrent or sequenced therapy)	40^a^ 20^b^	90.5% of patients with a response had an ongoing response at time of analysis (6.1 to 72.1 weeks)	53%^a^ 18%^b^	Nil Nil	NR	NR

^a^Concurrent therapy; ^b^Sequenced therapy; NR: not reported.

## Abbreviations

BRAF: B-Raf protein
BRAFi: BRAF inhibitor
CR: Complete response
CTLA-4: Cytotoxic T lymphocyte-associated antigen 4
ERK: Extracellular signal-regulated kinase
FDA: Food and Drug Administration
HR: Hazard ratio
MAPK: Mitogen-activated protein kinase
MHC: Membrane histocompatibility complex
mTOR: Mammalian target of rapamycin
NR: Not reported
ORR: Overall response rate
OS: Overall survival
PD-1: Programmed cell death 1
PD-L1: Programmed cell death 1 ligand 1
PFS: Progression-free survival
PI3K: Phosphoinositide 3-kinase
PR: Partial response
SCC: Squamous cell carcinoma
TCR: T cell receptor
V600: Amino acid substitution at position 600 in BRAF from valine
V600E: Amino acid substitution at position 600 in BRAF from valine to glutamic acid
V600K: Amino acid substitution at position 600 in BRAF from valine to lysine.
